# Runs of Homozygosity Uncover Potential Functional-Altering Mutation Associated With Body Weight and Length in Two Duroc Pig Lines

**DOI:** 10.3389/fvets.2022.832633

**Published:** 2022-03-08

**Authors:** Xiaopeng Wang, Guixin Li, Donglin Ruan, Zhanwei Zhuang, Rongrong Ding, Jianping Quan, Shiyuan Wang, Yongchuang Jiang, Jinyan Huang, Ting Gu, Linjun Hong, Enqin Zheng, Zicong Li, Gengyuan Cai, Zhenfang Wu, Jie Yang

**Affiliations:** ^1^College of Animal Science and National Engineering Research Center for Breeding Swine Industry, South China Agricultural University, Guangzhou, China; ^2^Guangdong Wens Breeding Swine Technology Co., Ltd., Yunfu, China; ^3^Guangdong Provincial Laboratory of Lingnan Modern Agricultural Science and Technology, Guangzhou, China

**Keywords:** Duroc pigs, runs of homozygosity, candidate genes, missense mutation, association analysis

## Abstract

Runs of homozygosity (ROH) are widely used to investigate genetic diversity, demographic history, and positive selection signatures of livestock. Commercial breeds provide excellent materials to reveal the landscape of ROH shaped during the intense selection process. Here, we used the GeneSeek Porcine 50K single-nucleotide polymorphism (SNP) Chip data of 3,770 American Duroc (AD) and 2,096 Canadian Duroc (CD) pigs to analyze the genome-wide ROH. First, we showed that AD had a moderate genetic differentiation with CD pigs, and AD had more abundant genetic diversity and significantly lower level of inbreeding than CD pigs. In addition, sows had larger levels of homozygosity than boars in AD pigs. These differences may be caused by differences in the selective intensity. Next, ROH hotspots revealed that many candidate genes are putatively under selection for growth, sperm, and muscle development in two lines. Population-specific ROHs inferred that AD pigs may have a special selection for female reproduction, while CD pigs may have a special selection for immunity. Moreover, in the overlapping ROH hotspots of two Duroc populations, we observed a missense mutation (rs81216249) located in the growth and fat deposition-related supergene (*ARSB*-*DMGDH*-*BHMT*) region. The derived allele of this variant originated from European pigs and was nearly fixed in Duroc pigs. Further selective sweep and association analyses indicated that this supergene was subjected to strong selection and probably contributed to the improvement of body weight and length in Duroc pigs. These findings will enhance our understanding of ROH patterns in different Duroc lines and provide promising trait-related genes and a functional-altering marker that can be used for genetic improvement of pigs.

## Introduction

Runs of homozygosity (ROH) are defined as a contiguous genome segment of the identical haplotype inherited from a common ancestor without recombination (1). ROH fragments are widely distributed in human and livestock genomes, and the patterns of ROH length and frequency distribution in the genome are mainly attributed to demographic history and selection (1, 2). The short ROH fragment indicates ancestral inbreeding, while the longer ROH fragment reflects recent close inbreeding (3). Hence, ROH are considered as an advanced method for assessing the degree of inbreeding (*F*_ROH_) in individuals and populations, providing support for the true level of homozygosity (4). ROH patterns are not randomly distributed throughout the genome. Selection may strongly affect the distribution of ROH, and regions of genomic loci under selection tend to generate a high frequency of ROH (hotspots) (5). An increasing number of studies have confirmed that ROH hotspots are due to positive selection for economically important traits in cattle (6, 7), pigs (8–10), chickens (11, 12), goats (13, 14), and sheep (15, 16). Therefore, the identification and characterization of ROH in a population can provide new insights for uncovering the demographic history and the genetic basis of germplasm characteristics.

Duroc pigs were first developed in North America in the 1860's and have spread all over the world as one of the best-known lean pig breeds (17). Duroc pigs have the characteristics of fast growth rate, high resistance to adversity, and good carcass performance. However, due to the unspectacular female reproductive performance, Duroc pigs are currently used as terminal sires in breeding programs (18). In the past 100 years, Duroc pigs have been widely imported into many countries and bred into different lines with different features according to the preferences of breeders. From the perspective of selection, intense selection reduced the diversity of haplotype, and different selective sweeps contributed to the formation of germplasm characteristics in Duroc pigs (19). For example, numerous studies (20–27) have used the methods of selection signatures to reveal a lot of candidate genes related to growth, immunity, meat, and carcass quality traits of Duroc pigs. Currently, the ROH pattern has been used to detect genetic diversity and genomic regions putatively under strong selection in Duroc pigs. For instance, Schiavo et al. (10) used the 60 K single-nucleotide polymorphism (SNP) data of 573 Italian Duroc pigs to reveal the distribution of ROH and *F*_ROH_. They also used the 80 K SNP data of 48 Italian Duroc pigs to detect an ROH island including genes that have been shown to affect body size (28). Grossi et al. (29) evaluated the *F*_ROH_ using the 60 K SNP data of 1,066 Canadian Duroc pigs. Gorssen et al. (30) found that the incidence of SNPs in a number of ROH hotspots of the Duroc genome was higher than 80%, and genes associated with coat color, blood physiology, and body size traits were putatively under intensive selection. Nevertheless, few studies have compared ROH patterns between different Duroc pig lines to reveal the potential differences in selection or breeding processes between lines. The aim of this study was to identify the distribution of ROHs in the genomes of 3,770 American Duroc (AD) and 2,096 Canadian Duroc (CD) pigs. Then, ROH hotspots were detected to reveal the different selection directions and the potential causal mutation related to the body size traits of the two Duroc lines.

## Methods

### Ethics Statement

All animal experiments used in this study were in accordance with the guidelines of the Regulations for the Administration of Affairs Concerning Experimental Animals (Ministry of Science and Technology, China, revised June 2004) and approved by the Animal Care and Use Committee of South China Agricultural University, Guangzhou, China (SCAU#2013-10).

### Animals, Genotyping, and Quality Control

In this study, ear tissue samples of 3,770 AD pigs (2,280 males and 1,490 females) and 2,096 CD pigs (1,017 males and 1,079 females) were collected from two core breeding farms of Wens Foodstuffs Group Co., Ltd. (Guangdong, China) between 2013 and 2017. During the fattening period of 30–100 kg body weight, all pigs in the two groups maintained uniform feeding conditions, fine fodder, and consistent management to minimize the influence of non-genetic factors (31). The genomic DNA was extracted from ear samples following the standard phenol/chloroform method. Genotyping was performed using the GeneSeek Porcine 50 K SNP Chip, which contains 50,703 genomic SNP markers. Quality control was conducted using PLINK v1.90 software (32) under the following criteria: (1) call rates of SNPs and individuals higher than 90%; (2) the Hardy–Weinberg equilibrium higher than 10^−6^; (3) all unmapped SNPs and those on sex chromosomes were discarded; and (4) the minor allele frequency (MAF) was not set since pruning low MAF may ignore a large number of homozygous regions (10). After quality control, a set of all 5,866 individuals and 45,424 SNPs was retained for subsequent analyses.

### Analysis of Genetic Diversity

In this section, MAF was set as 0.01, leaving 5,866 individuals and 39,416 SNPs for genetic diversity analysis. PLINK v1.90 software (32) was used to calculate the expected heterozygosity (He) and observed heterozygosity (Ho). Effective population size (*N*_e_) was estimated by *SNeP* software (33) with linkage disequilibrium (LD) method. The formula as follows: *N*_*e*(*t*)_ = $\;{\left(4f\left(C_{t}\right)\right)}^{-1}\left({E\left[r_{adj}^{2}|C_{t}\right]}^{-1}-\alpha \right)$, where *N*_*e*(*t*)_ is the *N*_e_ *t* generations ago, *C*_*t*_ is the recombination rate t generations ago inferred by the method of Sved and Feldman (34), $E\left[r_{adj}^{2}|C_{t}\right]$ is the LD expectancy adjusted for sampling bias, *f*(*C*_*t*_) is a modified function of the recombination rate based on the genetic distances with default value of 1 Mb = 1 cM, and α is a constant. Finally, nucleotide diversity (π) and fixation index (*F*_ST_) within and between AD and CD pigs were calculated by VCFtools (35) software. As individual π and *F*_ST_ values may be subjected by genotyping and missing errors in chip data, consistent with previous literature (36, 37), we estimated these statistics using a 500-kb sliding window.

### Detection and Classification of Runs of Homozygosity

ROHs were detected for each individual using PLINK v1.90 software (32) by sliding window method on the genome. The following parameters were used to define ROHs (8–10): (1) the minimum length of ROHs was 1 Mb; (2) a sliding window of 50 SNPs across the genome; (3) each window allowed one heterozygous genotype and five missing SNPs to avoid false negatives caused by occasional genotyping errors and missing genotypes; (4) the maximum gap between continuous SNPs was 1 Mb; (5) the required minimum SNP density was set to 100 kb; and (6) each ROH contained at least 63 and 68 consecutive SNPs in AD and CD pigs, respectively, which were computed by the following formula (38):


$$\begin{array}{l} l=\frac{\ln\frac{\alpha }{n_{s}\times n_{i}}}{\ln\left(1-\bar{het}\;\right)}. \end{array}$$

Where α is the percentage of false positive ROHs, which was set 0.05 in this study, *n*_*s*_ is the number of SNPs per individual, *n*_*i*_ is the number of individuals, and $\bar{he}t$ is the proportion of heterozygosity across all SNPs. In this study, the detected ROHs were divided into three categories for further analyses (39): 1–5, 5–10, and >10 Mb. Finally, we calculated and compared the numbers of each ROH length category and the ratio of ROH on each autosome of the two Duroc lines.

### Estimation of Inbreeding Coefficient

Two methods of genomic inbreeding coefficient for each individual were calculated using PLINK v1.90 software (32): (1) SNP-based inbreeding coefficient (*F*_HOM_) was estimated with the command set to “–het.” (2) ROH-based inbreeding coefficient (*F*_ROH_) was assessed for each individual according to McQuillan et al. (40) as follows: *F*_ROH_ = *L*_ROH_ / *L*_auto_, where *L*_ROH_ is the total size of ROHs in the genome of each individual and *L*_auto_ is the total size of 18 autosomes of pigs covered by SNPs, which was 2.45 Gb (41). We estimated the *F*_ROH_ of total sizes, 1–5, 5–10, and >10 Mb. Pearson correlation analyses were calculated among different *F*_ROH_ classifications and *F*_HOM_ in two Duroc populations.

### Detection of ROH Hotspots and Coldspot

The percentage of SNP occurrences in ROHs was calculated to characterize the genomic regions of ROH hotspots. In previous studies (7, 8, 11, 15, 42), the threshold of ROH hotspots was usually set as the top 1% and 5% of the SNP occurrences. For a better comparison, similar to a previous study (30), we defined the ROH hotspots with a frequency of SNP occurrences exceeding 80% (top 1.96% in AD pigs and top 1.46% in CD pigs) as significant regions putatively under selection. In addition, we retrieved the 50 K SNP data of 11 European wild boars (EWB) from a previous study (43). Following the quality control mentioned above (MAF > 0.01), a total of 34,196 SNPs remained. Then, the values of π, Tajima's *D*, and *F*_ST_ among AD, CD, and EWB pigs were calculated using VCFtools software (35) within 500-kb sliding bins to validate the candidate regions under selection. In addition to ROH hotspot, regions without ROH in any of the animals were considered ROH coldspots (44). These regions might be produced by high recombination rates and are likely enriched for variants with severe adverse effects on fitness in homozygotes (45).

### Candidate Gene, Pathway, and Functional Analyses

In this study, the average distance between SNPs in the quality control data used to detect ROH was 53.9 kb, and the minimum density of SNPs in ROH was 100 kb. In addition, a strong LD typically extends up to about 100 kb in the pig genome (46). Therefore, candidate genes were annotated *via* the *Ensembl* database (*Sscrofa* 11.1, http://www.ensemble.org/) at 100-kb regions (upstream 50 kb and downstream 50 kb) flanking the SNPs of ROH hotspots. The Gene Ontology (GO) terms and Kyoto Encyclopedia of Genes and Genomes (KEGG) pathways were analyzed for all candidate genes by *Metascape* database (https://metascape.org/). Meanwhile, in order to reveal the relevance of selected regions to Duroc quantitative traits, ROH hotspots were mapped and compared with the pig quantitative trait loci (QTL) database (https://www.animalgenome.org/cgi-bin/QTLdb/SS/index, release 45). We downloaded the pig QTL file and discarded QTLs with uncertain positions and length more than 1 Mb (26), resulting in 21,952 informative QTLs for analysis.

### Identification of Putative Functional SNPs and Their Relationship With Production Traits

The candidate SNPs first predicted the effects on protein function using the Sorting Intolerant From Tolerant (SIFT) score *via* the Variant Effect Predictor (VEP) program in *Ensembl* database (47). SNPs with SIFT scores of ≤ 0.05 and >0.05 were predicted to be deleterious and tolerant (48), respectively. The *ARSB*-*DMGDH-BHMT* region [chromosome (SSC) 2:87,614,952–87, 935,253, nine SNPs, including a putative deleterious mutation (rs81216249)] was retrieved from the publicly available 60 K SNP data of 1,522 pigs from 107 global populations (49). The dataset includes Asian domestic pigs, Asian wild boars, Western commercial pigs (including Duroc, Large White, Landrace, Berkshire, Hampshire, and Pietrain pigs), European domestic pigs, European wild boars, African feral pigs, American feral pigs, Oceania feral pigs, and outgroup populations. Then, the haplotypes of nine SNPs were constructed using fastPHASE software (50) in global pig populations. In order to uncover the evolutionary history of the missense mutation (rs81216249) and its potential effect on the phenotype in Duroc pigs, we calculated the allele frequency of this variant in global pig breeds. Next, we downloaded the publicly available genotyping (80 K SNPs) and phenotypic (body weight and body length) data of 365 Sujiang sows (a synthetic breed derived from Chinese Jiangquhai, Fengjing, and Western Duroc pigs) from a previous study (51). The association analysis between this missense mutation and body weight and body length was implemented using “–linear” command in PLINK (32) with age and batch as covariates. The linear model is as follows: *y*_*ijkl*_ = μ+*SNP*_*j*_+*COV*1_*k*_+*COV*2_*l*_+*e*_*ijkl*_, where *y*_*ijkl*_ is the body weight or body length phenotype of the *i-th* individual, μ is the mean term for the body weight and body length, respectively, *SNP*_*j*_ is the fixed effect with three levels (GG, GA, and AA coding as 0, 1, and 2), *COV*1_*k*_ is the fixed effect for age, *COV*2_*l*_ is the fixed effect of batch, and *e*_*ijkl*_ is the random residual.

## Results

### Estimation of Genetic Diversity and Inbreeding Coefficient

**Figure 1:**
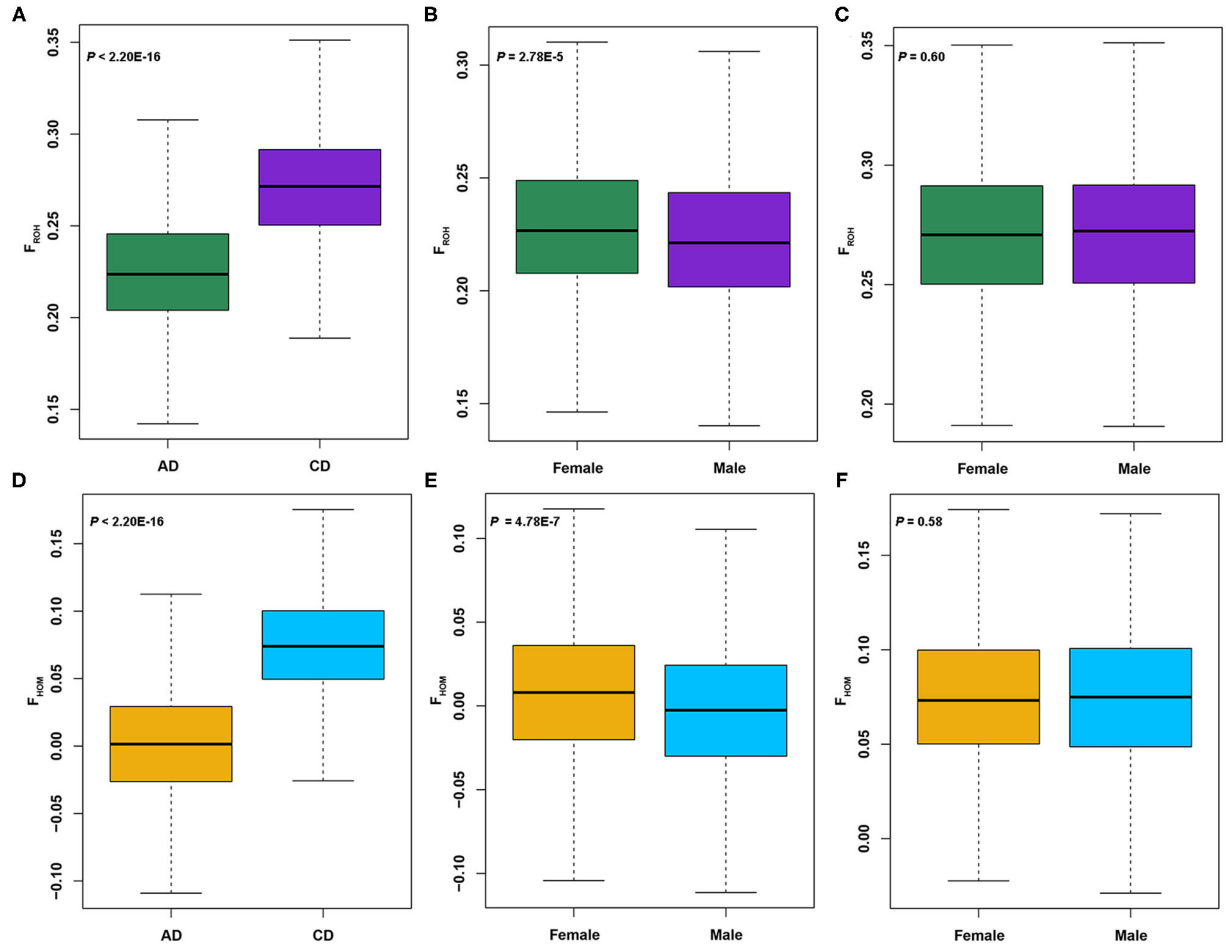
Analysis of the inbreeding coefficients of American Duroc (AD) and Canadian Duroc (CD) pigs based on single-nucleotide polymorphism (SNP) and runs of homozygosity (ROH). **(A)** *F*_ROH_ of two Duroc lines. **(B,C)** *F*_ROH_ in females and males of AD and CD pigs, respectively. **(D)** *F*_HOM_ of two Duroc lines. **(E,F)** *F*_ROH_ in females and males of AD and CD pigs, respectively.

Four metrics were calculated to evaluate the genetic diversities of two Duroc lines: Ho, He, Ne, and π. As seen in Table 1, the Ho (0.29), He (0.30), and π (5.22 × 10^−6^) values of AD pigs were greater than those of CD (Ho = 0.27, He = 0.28, and π = 4.83 × 10^−6^) pigs. Then, two inbreeding coefficients based on genomic data of each individual were computed. In our previous study (27), the *F*_ROH_ calculated using consecutive pattern showed that the *F*_ROH_ of AD pigs was significantly lower than that of CD pigs. At the present study, we used the sliding window method to estimate *F*_ROH_ and obtained the same result, and the average *F*_ROH_ (0.23) of AD was significantly (*p* < 2.20 × 10^−16^) less than that of CD (*F*_ROH_ = 0.27) pigs (Figure 1A). In addition, the average *F*_HOM_ (0.0025) of AD was also significantly (*p* < 2.20 × 10^−16^) lower than that of CD (*F*_HOM_ = 0.075) pigs (Figure 1D). Similar to previous studies (8, 19), we also found a highly significant positive correlation between *F*_HOM_ and *F*_ROH_ in AD (*r* = 0.89, *p* < 2.20 × 10^−16^) and CD (*r* = 0.84, *p* < 2.20 × 10^−16^) pigs (Supplementary Figure 1). Considering that Duroc pigs are mainly used as terminal male parents to cross with other commercial pigs to produce hybrid pigs, the selection intensity of boars may be higher than sows, which may result in higher levels of *F*_HOM_ and *F*_ROH_ in boars. Unexpectedly, in AD pigs, the values of *F*_HOM_ and *F*_ROH_ were significantly (*p* = 4.78 × 10^−7^ in F_HOM_ and *p* = 2.78 × 10^−5^ in *F*_ROH_) higher in females than in males (Figures 1B,E). In comparison, there was no difference in *F*_HOM_ and *F*_ROH_ between males and females of CD pigs (Figures 1C,F). The *F*_ROH_ values were divided into three classes, and the *F*_ROH> 10_ displayed the highest correlation coefficients with *F*_ROH_ in both AD (*r* = 0.91, *p* < 2.20 × 10^−16^) and CD (*r* = 0.91, *p* < 2.20 × 10^−16^) pigs (Supplementary Figure 1). According to the formula *L*_ROH_ = 100 / (2*g* × cM), where *g* is the generation ago, and 1 cM is approximately equal to 1 Mb (52). The ROHs were mainly accumulated within the last five generations in AD and CD pigs. The Ne was estimated from the LD method, and the results showed that AD (Ne = 99) was slightly larger than CD (Ne = 98) pigs (Table 1) in the last 13 generations. According to the classification by Hartl (53), the value of *F*_ST_ ranging from 0 to 0.05 indicates small genetic differentiation, 0.05 to 0.15 indicates moderate genetic differentiation, and > 0.15 indicates obvious genetic differentiation. The *F*_ST_ value between AD and CD pigs was 0.093, indicating a moderate degree of genetic divergence between two Duroc lines.

**Table 1:** Genetic diversity of Duroc pigs.

**Lines**	**He**	**Ho**	**π**	**Ne**	***F*_HOM_**	***F*_ROH_**	***F*_ROH1−5_**	***F*_ROH5−10_**	***F*_ROH10_**
AD	0.30	0.29	5.22 × 10^−6^	99	0.0025	0.23	0.045	0.055	0.13
CD	0.28	0.27	4.83 × 10^−6^	98	0.075	0.27	0.044	0.061	0.17

*AD, American Duroc pigs; CD, Canadian Duroc pigs; He, expected heterozygosity; Ho, observed heterozygosity; π, nucleotide diversity; Ne, effective population size; F_HOM_, SNP-based inbreeding coefficient; F_ROH_, runs of homozygosity based inbreeding coefficient; F_ROH1−5_, F_ROH_ of 1–5 Mb category; F_ROH5−10_, F_ROH_ of 5–10 Mb category; F_ROH10_, F_ROH_ of > 10 Mb category*.

### Distribution of Runs of Homozygosity

**Figure 2:**
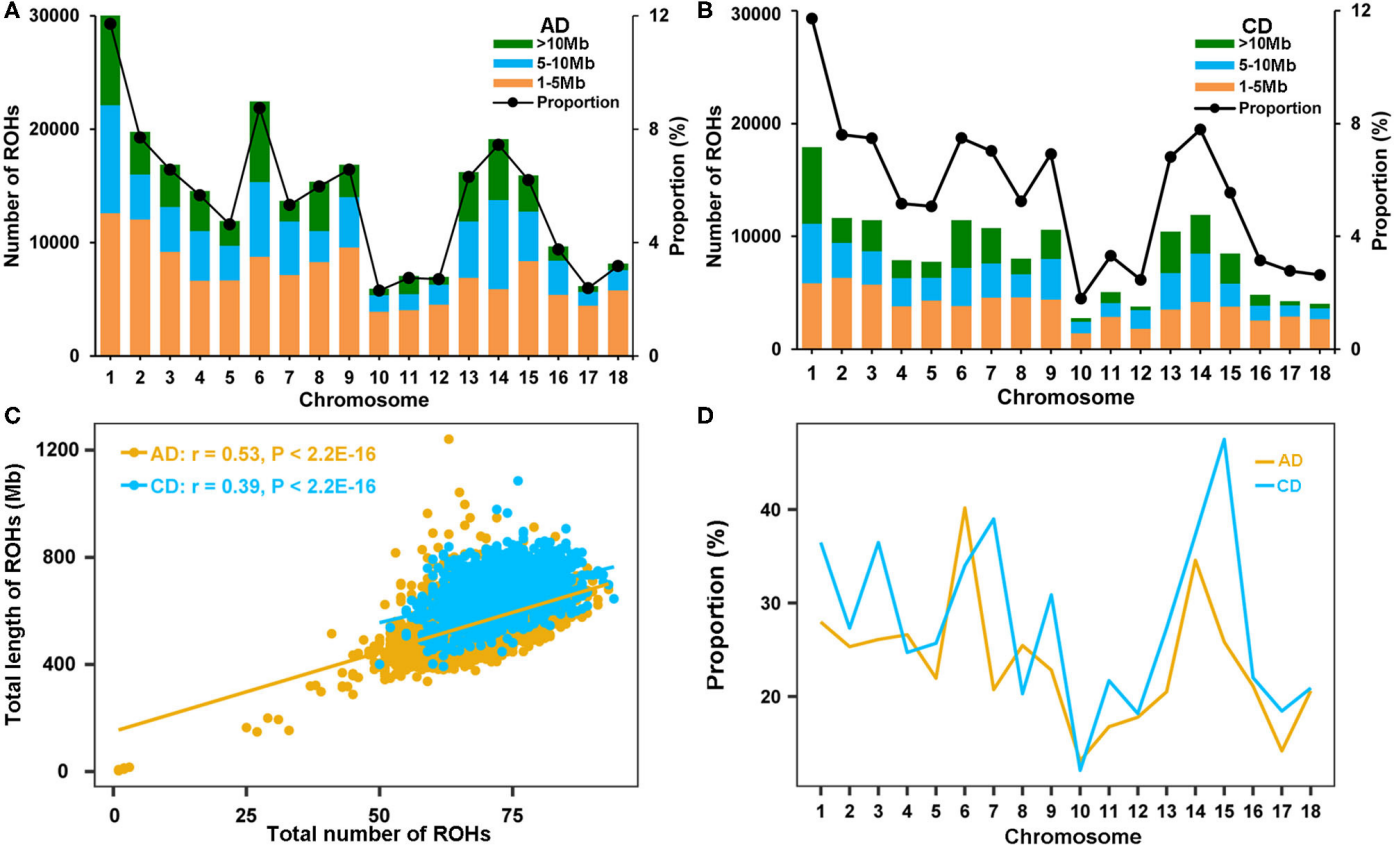
Summary results of ROH in two Duroc lines. **(A,B)** The number distribution of the three ROH length categories (bars) and average percentage of ROH to the total ROH length (lines) on each autosome of AD and CD pigs, respectively. **(C)** The total number of ROH and the total length of ROH, per animal, for AD and CD pigs. **(D)** The average percentage of ROH to chromosome length on each autosome of AD and CD pigs.

The genome-wide ROHs were assessed on 18 autosomes of all tested individuals. A sum of 256,530 and 152,877 ROHs were detected in AD and CD pigs, respectively (Supplementary Table 1). The average numbers of ROH were 68.05 and 72.94 in AD and CD pigs, respectively. The distribution of ROHs on chromosomes was uneven for the three length categories. The numbers of short fragment of ROH (1–5 Mb) took up the largest part of the total ROHs (49.04% in AD pigs and 43.81% in CD pigs). The smallest chromosome coverage of ROH by dividing the total ROH length was found on SSC10, and the largest was on SSC1 in both AD and CD pigs (Figures 2A,B). The number of ROH was significantly positively correlated with the length of each chromosome in AD (*r* = 0.93, *p* = 2.50 × 10^−8^) and CD (*r* = 0.91, *p* < 1.17 × 10^−7^) pigs. Although the number of ROH_1−5*Mb*_ was the largest, the proportion of ROH in this category to the total ROH length was not the largest (19.90% in AD pigs and 16.33% in CD pigs). In comparison, ROH fragments larger than 10 Mb had the smallest number (22.36% in AD pigs and 26.67% in CD pigs) but the largest proportion of the total ROH length (55.54% in AD pigs and 61.11% in CD pigs). The total ROH length of each AD pig ranged from 3.20 to 1,240.97 Mb, while each CD pig ranged from 392.64 to 1,085.34 Mb (Figure 2C and Table 2). AD (*r* = 0.53, *p* < 2.20 × 10^−16^) and CD (*r* = 0.39, *p* < 2.20 × 10^−16^) pigs had significant correlations between the number and total length of ROHs per animal (Figure 2C). The coverage of ROH on each autosome was also estimated to compare the distribution of ROH in autosomes. In general, CD pigs had a high genome coverage of ROH in most autosomes (except for SSC4, 6, 8, and 10) than AD pigs (Figure 2D).

**Table 2:** Distribution of ROH hotspots and coldspot in two Duroc lines.

**Population**	**Chromosome**	**Position (bp)**	**Length (Mb)**	**Number of SNPs**
**Hotspots**				
AD	2	86,008,048–89,123,545	3.11	73
	6	86,543,912–104,721,678	18.18	301
	6	106,257,182–116,228,067	9.97	114
	8	101,139,901–106,166,798	5.02	83
	14	74,735,219–80,125,142	5.39	103
	14	91,769,946–101,133,523	9.36	163
	14	114,883,471–118,110,675	3.23	52
CD	1	236,248,295–239,741,131	3.49	69
	2	23,734,384–24,878,655	1.14	36
	2	86,176,683–89,123,545	2.95	69
	3	46,520,543–55,461,707	8.94	146
	6	86,543,912–97,417,152	10.87	198
	7	51,398,162–54,185,548	2.79	35
	14	115,136,883–115,734,563	0.60	8
	14	121,544,610–122,473,672	0.93	26
	15	82,115,926–83,054,536	0.94	19
	15	102,554,592–107,134,695	4.58	56
**Coldspot**				
AD and CD	2	27,459–695,777	0.67	15

*AD, American Duroc pigs; CD, Canadian Duroc pig*.

### QTLs, Candidate Genes, and Functional Processes in ROH Hotspots and Coldspot

**Figure 3:**
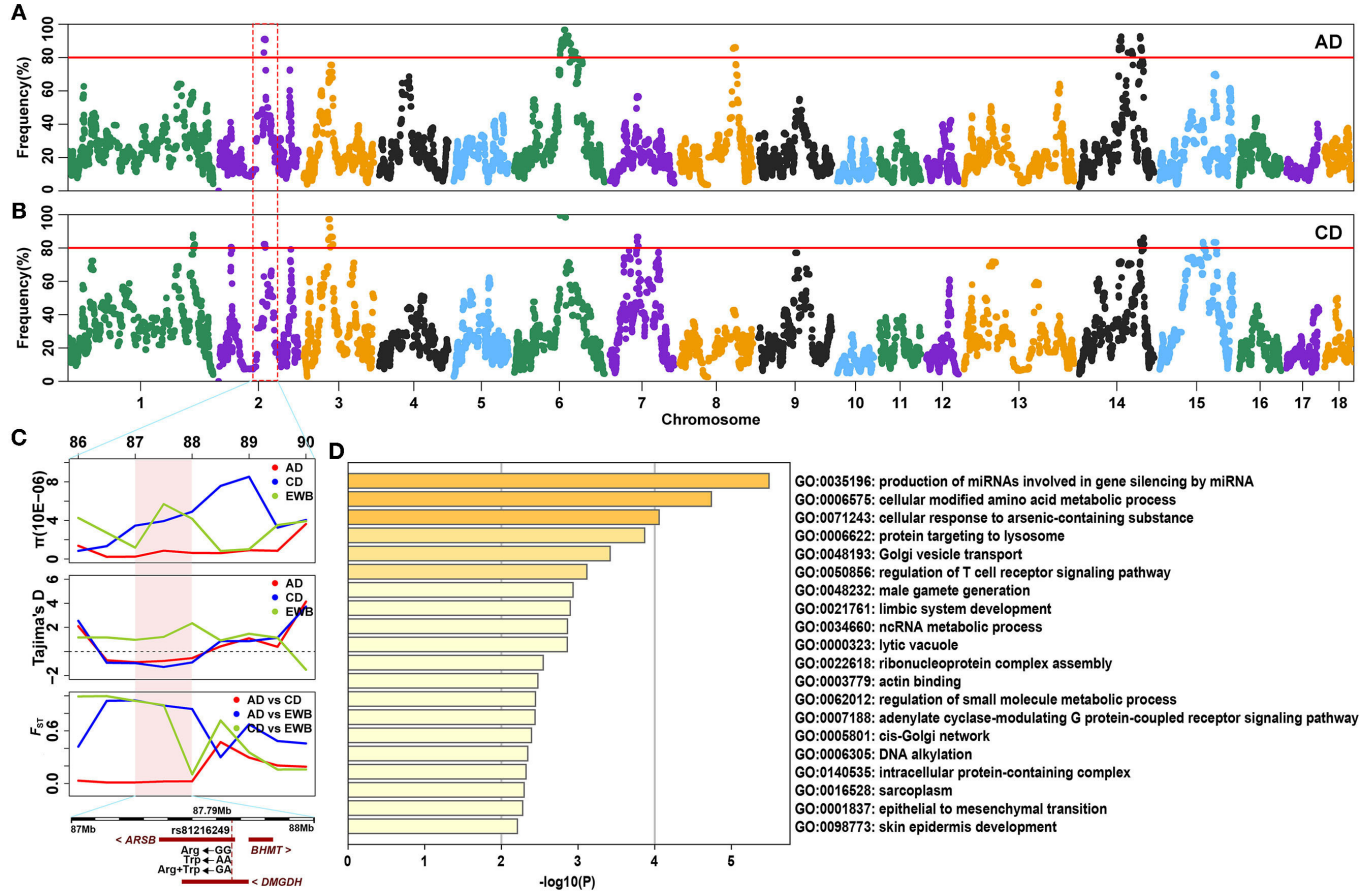
Detection of ROH hotspots in two Duroc lines. **(A,B)** Manhattan plot of the occurrence (%) of each SNP in ROHs of AD and CD pigs, respectively. The red lines correspond to the significance threshold (80%). **(C)** The identical ROH region (SSC2: 87.00–88.00 Mb) was detected by π, Tajima's *D*, and *F*_ST_ methods in AD and CD pigs. The significant region is colored by a pink background. **(D)** Top 20 significant Gene Ontology (GO) terms of overlapping genes identified by ROH.

SNPs with an occurrence frequency higher than 80% in ROH were considered as candidate loci putatively under selection. In AD pigs, seven ROH hotspots were detected on four chromosomes, including 891 SNPs with a total length of 18.18 Mb. The highest number (*n* = 301) and occurrence frequency (92.79%) of SNPs were found on SSC6 (Figure 3A and Table 2). A total of 259 candidate genes resided in 100-kb regions surrounding the 891 SNPs (Supplementary Figure 2). For CD pigs, 10 ROH hotspots were detected, containing 663 candidate SNPs. A total of 271 candidate genes were annotated in 100-kb regions surrounding these 663 SNPs (Supplementary Figure 2). Among those ROH hotspots, the highest number (*n* = 198) and occurrence frequency (100.00%) of SNPs were also identified on SSC6 (Figure 3B and Table 2). A total of three overlapping ROH hotspots (on SSC2, 6, and 14, including 275 SNPs) were identified in AD and CD pigs (Figure 3 and Supplementary Figure 2). Eighty-six QTLs (41 terms) were identified in the overlapping ROH hotspots of AD and CD pigs (Supplementary Table 2). These QTLs were mainly enriched in health (“mean corpuscular volume and platelet count”), exterior (“splay leg”), meat and carcass (“capric acid content”), and production (“days to 110 kg” and “feed conversion ratio”) traits. A total of 120 overlapping genes were mainly enriched in GO terms of metabolic, muscle development, reproduction, immunity, and behavior (Figure 3D and Supplementary Table 3). Among these genes, three genes were related to sperm development (*PUM1, CFAP43*, and *TEKT2*), six genes (*DMGDH, ARSB, BHMT, MATN1, MFSD2A*, and *ZMPSTE24*) were involved in growth, and two genes (*HOMER1* and *AKIRIN1*) were relevant to muscle development (Table 3). Meanwhile, ROH hotspots generally showed the low level of π values, Tajima's *D* < 0, high values of *F*_ST_ between Duroc and EWB pigs, and low values of *F*_ST_ between two Duroc lines, such as SSC2 (86,176,683-89,123,545) and SSC6 (86,543,912-97,417,152) (Figure 3C and Supplementary Figure 3).

**Table 3:** Some candidate genes related to phenotypic traits in ROH hotspots and coldspot.

**Gene^**a**^**	**Chromosome**	**Position (Mb)**	**Population**	**Gene function**
**Hotspots**				
*DMGDH*	2	87.68–87.87	AD, CD	Growth
*ARSB*	2	87.61–87.83	AD, CD	Body weight and skeletal development
*BHMT*	2	87.87–87.94	AD, CD	Body weight and fat deposition
*HOMER1*	2	88.12–88.26	AD, CD	Muscle development
*MATN1*	6	87.34–87.35	AD, CD	Skeletal development
*PUM1*	6	87.55–87.69	AD, CD	Sperm development
*AKIRIN1*	6	94.89–94.91	AD, CD	Muscle development
*TEKT2*	6	92.23–92.24	AD, CD	Sperm development
*MFSD2A*	6	95.74–95.81	AD, CD	Growth
*ZMPSTE24*	6	96.01–96.06	AD, CD	Growth
*CFAP43*	14	115.07–115.17	AD, CD	Sperm formation
*PSMA8*	6	110.8–110.84	AD	Sperm development
*FGF2*	8	101.28–101.34	AD	Skeletal development
*ADAD1*	8	101.54–101.72	AD	Sperm development
*MAD2L1*	8	103.93–103.94	AD	Embryonic viability
*SEC24D*	8	104.81–105.01	AD	Embryonic viability
*MYOZ1*	14	76.44–76.45	AD	Growth
*CSTF2T*	14	98.11–98.11	AD	Sperm development
*SGMS1*	14	99.15–99.46	AD	Sperm development
*SIT1*	1	236.37–236.37	CD	Immunity
*RECK*	1	236.82–236.90	CD	Skeletal development
*XPA*	1	239.53–239.57	CD	Growth and weight
*TRAF6*	2	24.60–24.63	CD	Growth
*FHL2*	3	49.36–49.47	CD	Skeletal development
*TSGA10*	3	55.18–55.32	CD	Sperm development
*REV1*	3	54.14–54.86	CD	Growth and weight
*RANBP2*	3	47.53–47.60	CD	Growth and weight
*IL1R1*	3	52.19–52.29	CD	Body weight
*MAN2A2*	7	53.50–53.52	CD	Sperm formation
*SATB2*	15	102.96–103.15	CD	Skeletal development
*ICA1L*	15	106.3–106.38	CD	Sperm development
*CD28*	15	107.13–107.16	CD	Immunity
**Coldspot**				
*RIC8A*	2	0.03–0.39	AD, CD	Embryonic viability
*PTDSS2*	2	0.26–0.28	AD, CD	Testicular development
*RNH1*	2	0.28–2.90	AD, CD	Embryonic viability

a *Some candidate genes associated with interesting phenotypic traits; AD, American Duroc pigs; CD, Canadian Duroc pigs*.

In addition to the overlapping ROH hotspots, we also detected several population-specific ROH regions in two Duroc lines (Table 3). For AD pigs, a total of 108 (57 terms) AD-specific QTLs were retrieved for the QTLs of ROH hotspots (Supplementary Table 2). These QTLs were mainly enriched in exterior (“thoracolumbar vertebra number” and “iris pigmentation”), meat and carcass (“backfat between 3rd and 4th last ribs”), and reproduction (“age at puberty” and “number of stillborn”) traits. A total of 138 AD-specific genes were used to perform the GO and KEGG analyses. The results showed that candidate genes were mainly enriched in metabolic, muscle development, immunity, and organ development (Supplementary Table 3). Among these candidate genes, two genes (*ADAD1* and *PSMA8*) were related to sperm development, two genes (*MAD2L1* and *SEC24D*) were involved in embryonic development, and two genes (*FGF2* and *MYOZ1*) were associated with growth (Table 3). For CD pigs, 479 (43 terms) CD-specific QTLs were identified (Supplementary Table 2), and these QTLs were mostly enriched in health (“CD8-positive leukocyte percentage and basophil percentage”) and exterior (“coping behavior”) traits. The GO and KEGG analyses revealed that 151 CD-specific candidate genes were mainly enriched in immunity, stress response, and metabolism (Supplementary Table 3). Among these candidate genes, three genes (*TSGA10, MAN2A2*, and *ICA1L*) were associated with sperm development, eight genes (*RECK, XPA, TRAF6, FHL2, REV1, RANBP2, IL1R1*, and *SATB2*) were relevant to growth and/or skeletal development, and two genes (*SIT1* and *CD28*) were related to immunity. In addition, we also found low values of π and Tajima's *D* and high values of *F*_ST_ between AD/CD and EWB in population-specific ROH regions, for example, SSC8 (103,000,000-106,166,798) in AD pigs and SSC1 (236,248,295-237,000,000) and SSC3 (49,360,000-55,461,707) in CD pigs (Supplementary Figure 3).

Interestingly, an overlapping region with 15 SNPs was observed on SSC2 (0.027–0.70 Mb) of the two Duroc lines, which did not occur in any ROH (ROH coldspot). Three reproduction-related genes (*RIC8A, PTDSS2*, and *RNH1*) were annotated to this 0.67 Mb region (Table 3). Previous study showed that ROH coldspots were considered to have loci with the capability of avoiding purely lethal or cryptic mutation-critical functions (45). Therefore, this coldspot region may play an important role in the viability of Duroc pigs.

### Functional Annotation of Variants

**Figure 4:**
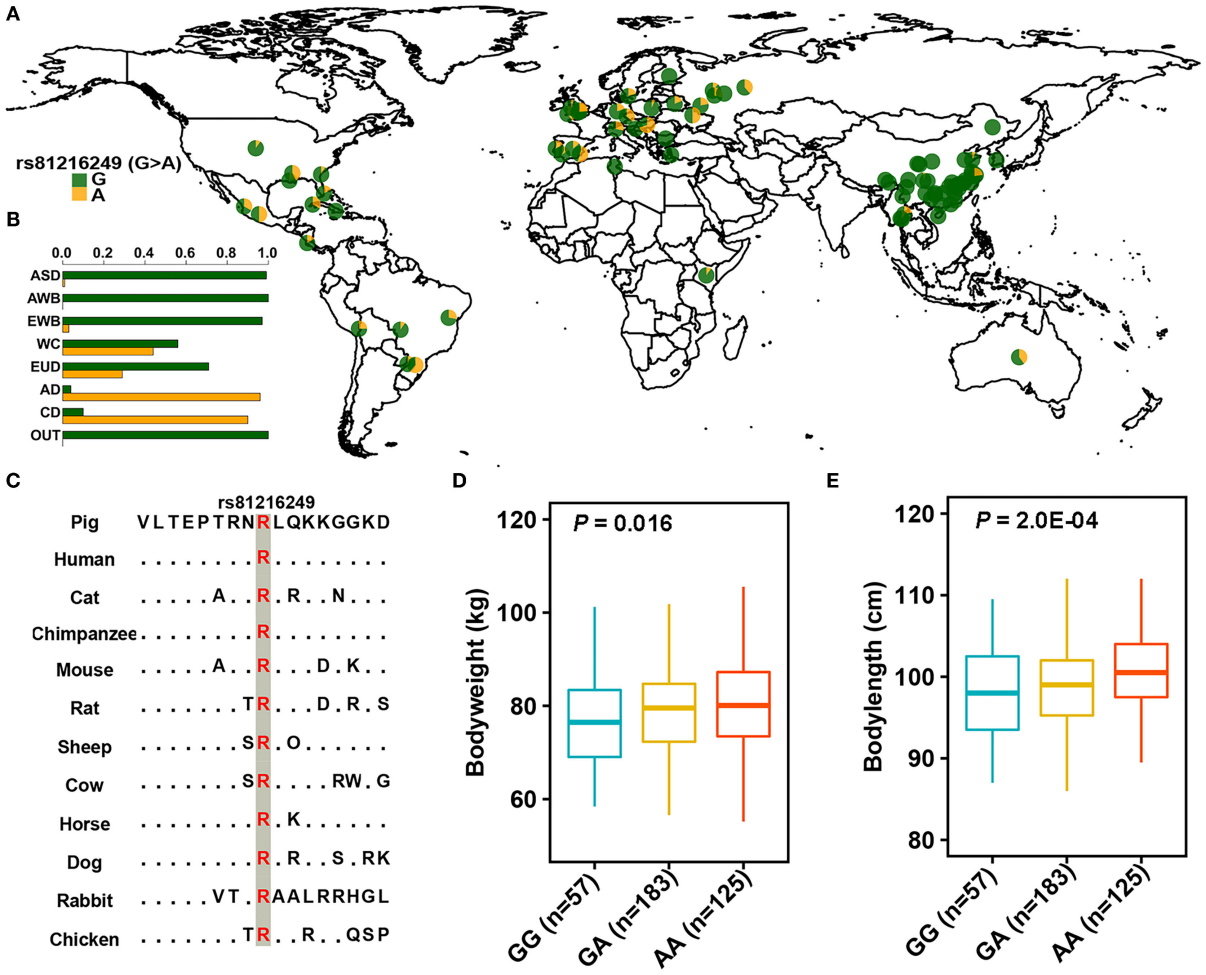
The distribution of rs81216249. **(A,B)** The allele frequency distribution of rs81216249 in global pig breeds. G and A denote ancestral and derived genotypes and are marked by green and yellow, respectively. ASD, AWB, EWB, EUD, WC, AD, CD, and OUT represent Asian domestic pigs, Asian wild boars, European wild boars, European domestic pigs, Western commercial pigs, American Duroc pigs, Canadian Duroc pigs, and outgroup populations, respectively. **(C)** Multispecies alignment of the protein sequences around the variant. **(D,E)** Association analysis of rs81216249 with body weight and body length in Sujiang pigs.

To better understand the genetic basis of ROH hotspots, we performed variant annotation on candidate loci. A total of 21 and 30 non-synonymous variants were observed in the ROH hotspots of AD and CD pigs (Supplementary Table 4), respectively. Notably, only one identical missense variant (rs81216249, g. 87792989G > A) was predicted as a functional-altering mutation (SIFT = 0) in AD and CD pigs. Five outgroup populations (*Babyrousa babyrussa, Phacochoerus africanus, Sus barbatus, Sus celebensis*, and *Sus verrucosus*) (49) were used to infer that G was an ancestral allele and A was a derived allele. This variant was highly conserved across multiple vertebrate species (Figure 4C). The frequency of derived allele (DAF) of this variant displayed a large difference between Duroc and other global pig breeds (Figures 4A,B and Supplementary Table 5). The DAF was near fixation in AD (96%), CD (90%), and other (80–95%) Duroc populations with unknown origin (49). The *F*_ST_-based neighbor-joining tree (54) revealed that these Duroc populations had different origins from AD and CD pigs (Supplementary Figure 4). This indicated that a high DAF of this mutation may present in Duroc pig breed. Intriguingly, the derived allele was at high prevalence in Western commercial and European domestic pigs but nearly absent in Chinese indigenous pigs [except for three hybrid breeds, Sutai, Licha, and Neijiang pigs, which were reported to be admixed with Duroc pigs (49)] (Figure 4A and Supplementary Table 5). This variant is located in the overlapping region of three genes (*ARSB*-*DMGDH*-*BHMT*, SSC2:87614952-87935253) and as a missense mutation of *DMGDH*. The values of π, Tajima's *D*, and *F*_ST_ revealed that the region of three genes was under positive selection (Figure 3C). The haplotype heat map of this region showed that most Duroc pigs had the same haplotype, revealing that this region was under heavy selection (Supplementary Figure 5). The cluster of supergenes (*ARSB*-*DMGDH*-*BHMT*) has an important role in growth and fat deposition traits (55). The above results indicated that this missense mutation may play an important role in the production traits of Duroc pigs. Finally, a correlation analysis was used to detect the effect of this functional-altering variant on the phenotypes of Sujiang pigs. The results showed that rs81216249 was significantly associated with body weight (*p* = 0.016) and body length (*p* = 0.0002). The phenotypic value of pigs with the AA genotype was higher than that of pigs with the GG and GA genotypes (Figures 4D,E).

## Discussion

### Artificial Selection Possibly Caused the Differences in Genetic Diversity and Inbreeding Levels of the Two Duroc Lines

In our study, combining the results of six genetic diversity and inbreeding indices (Ho, He, π, Ne, *F*_HOM_, and *F*_ROH_), AD pigs had an abundant genetic diversity and a low level of inbreeding than CD pigs. Similar to previous studies (8, 56), the values of *F*_ROH_ were larger than *F*_HOM_ in two Duroc populations. This may be caused by the inability of the *F*_HOM_ to differentiate between IBD and IBS alleles (57). The moderate genetic differentiation (*F*_ST_ = 0.093) indicated that two Duroc lines possibly experienced different demographic, inbreeding, and selection histories. *F*_ROH>10_ made a major contribution to *F*_ROH_ of the two Duroc lines, revealing the recent reduction in genetic diversity and increase in homozygosity in two Duroc lines. Due to the differences in computational methods and parameters, tested populations, sample sizes, and SNP arrays, the Duroc populations in this study displayed different ROH numbers and lengths compared with previous studies of ROH in Duroc pigs (10, 28–30). Two Duroc populations had low degrees of *F*_HOM_, likely due to the two Duroc populations raised in the same company (Wen's Foodstuff Group Co., Ltd.), which had the better inbreeding control and breeding programs. Our previous study (58) reported that LD decays faster with distance in AD than in CD pigs, implying that CD has a greater selective strength than AD pigs. A previous study (45) showed that, compared with medium and small ROH, recent strong directional selection may have a greater impact on long ROH, because it tends to produce long haplotypes. Based on the above results, we believe that the existence of genetic differences between the two Duroc lines may be more partly due to recent selection. Moreover, compared to CD pigs, the *F*_HOM_ and *F*_ROH_ values of females were larger than males in AD pigs. A reasonable explanation is that the traits associated with female reproduction may be selected to improve the poor fertility of AD pigs.

### Overlapping ROH Hotspots Uncovered the Same Breed Characteristics of the Two Duroc Lines

ROH patterns were mainly shaped by population bottlenecks, inbreeding, genetic drift, and intensive natural and artificial selection (3). Peripolli et al. (2) suggested that a lot of ROHs were shared among livestock individuals, which may be due to selection rather than just demographic history. Duroc pig is a representative commercial pig breed that has recently been strongly selected for economic traits. Sonesson et al. (59) reported that genomic selection may also lead to the risk of long homozygous segments appearing around QTL regions related to any given trait in the populations. Therefore, the investigation of ROH islands can provide information about selection signatures that are putatively derived from various selection events, directions, and adaptations to different production systems (60). We expected that overlapping ROH regions and genes may have undergone directional selection in Duroc pigs, which contributes to their breed characteristics such as excellent growth rate, carcass traits, male reproductive capacity, and meat quality. The results of QTLs and GO and KEGG analyses were in line with this expectation, and QTLs and functional processes were more enriched in health, production, and behavior. We also observed three sperm-related genes, six growth-related genes, and two muscle development-related genes putatively under selection that are functionally related to these breed features. For instance, the *PUM1* gene mediates activation and apoptosis of spermatogonia and acts as a post-transcriptional regulator of spermatogenesis in the testis (61). *TEKT2* is associated with male sterility and abnormal sperm morphology and function in mice (62). Mice lacking *CFAP43* show multiple morphological abnormalities of the flagella and impaired sperm motility (63). *MATN1* is expressed in cartilage structures such as the trachea, nasal septum, auricle, and epiphysis (64). *MFSD2A* knockout mice exhibit a smaller and leaner body size (65). Mice lacking the *ZMPSTE24* gene show premature signs of aging such as reduced body weight and subcutaneous fat, spinal prolapse, hair loss, and premature death (66). *HOMER1* (67) and *AKIRIN1* (68) are related to muscle development. Some candidate genes have been reported in previous selective sweep studies, such as *HOMER1* (23, 24) and *ARSB* (25). These promising genes associated with economic traits may contribute to the genetic breeding process of pigs.

In addition, we focused on neighboring genes (*ARSB*-*DMGDH*-*BHMT*) located on the overlapping ROH hotspots (SSC2:87614952-87935253). *ARSB* is related to abnormal morphology of the head, nose, and tail vertebra; fat/triglyceride levels; and reduced body size at birth and adulthood in mice (69). *ARSB* is also the causal gene of human mucopolysaccharidosis type VI (Maroteaux–Lamy syndrome), which is related to facial deformities and short stature (70). *DMGDH* plays an important role in regulating the insulin-like growth factor/growth hormone (IGF-1/GH) pathway, which is an important regulator of vertebrate growth (71). *BHMT* is associated with body weight, fat deposition, and energy metabolism (55, 72). Three genes are genetically linked in most vertebrates, and these functionally relevant genes, in some cases called “supergene,” may play an influential role in the physiological processes that affect growth and fat deposition (55). The selective sweep analyses of π, Tajima's *D*, and *F*_ST_ confirmed that the supergene experienced strongly positive selection in Duroc pigs. Therefore, we believe that these three genes are important genes associated with the production traits of Duroc pigs, and it is worthwhile to further explore the genetic mechanism of their impact on the phenotypes.

### Population-Specific ROH Hotspots Revealed Potential Differences in Breeding Objectives of the Two Duroc Lines

Compared with overlapping ROH regions, we detected the population-specific ROH hotspots to elucidate the potential differences in the breeding goals of the two Duroc populations. Population-specific ROH regions generally displayed distinct patterns of π, Tajima's *D*, and *F*_ST_, which may reveal unique artificial selection in the two pig lines. However, some population-specific QTLs were associated with the same traits, such as “average daily gain,” “intramuscular fat content,” and “coping behavior.” The first two traits are very important to the pork industry and have been targeted for positive selection in Duroc breeding. Coping behavior is considered as the reaction behavior of pig to aversive environment (73). In recent years, the intensive feeding model has been greatly developed in the pig industry, which may lead to the selection of “coping behavior” (26). The results of GO and KEGG analyses were consist with QTLs, in which two sets of population-specific candidate genes were enriched in some functional processes with similar functions, such as immune response, metabolism, and development. Simultaneously, we also detected many candidate genes related to superior breed characteristics in two population-specific ROH hotspots, such as sperm development (*PSMA8, ADAD1, CSTF2T*, and *SGMS1* in AD pigs and *TSGA10, MAN2A2*, and *ICA1L* in CD pigs) and growth (*FGF2* and *MYOZ1* in AD pigs and *RECK, XPA, TRAF6, FHL2, REV1, RANBP2, IL1R1*, and *SATB2* in CD pigs) traits. The results revealed that CD pigs had more candidate genes related to growth than AD pigs, which may be caused by their stronger selection as mentioned above. These same QTLs, similar functional processes, and candidate genes revealed the possible polygenic basis of these economic traits in the two populations.

Besides the potentially polygenic basis, population-specific ROHs can also uncover some different characteristics in two populations. For example, QTLs associated with exterior and production were enriched in AD pigs, such as “thoracolumbar vertebra number” and “backfat between 3rd and 4th last ribs.” In contrast, most QTLs related to health were enriched in CD pigs, such as “CD8-positive leukocyte percentage” and “CD8-negative leukocyte percentage.” Candidate genes of AD pigs were more significantly enriched in metabolic process, and CD pigs were more significantly enriched in the immune system process, which was consistent with our previous study on the selection signatures of these two lines (27). We also observed two genes (*MAD2L1* and *SEC24D*) associated with embryonic development in AD pigs and two immune-related genes (*SIT1* and *CD28*) in CD pigs. The *MAD2L1* gene is related to embryonic viability (74). Mice lacking *SEC24D* show early embryonic lethality (75). *SIR1* knockout mice show increased susceptibility to autoimmune encephalitis (76), and *CD28* affects T-cell proliferation in mice (77). Based on the above results, we infer that AD pigs may have a specific selection for female fertility, which was in line with the result that females harbored larger values of *F*_ROH_ and *F*_HOM_ than males in AD pigs. A previous study (78) also showed that AD pigs had a lower number of stillborn and a higher rate of born alive than CD pigs in the first two parities. In comparison, CD pigs may have a specific selection for immunity, possibly because they need to possess excellent adaptability to the intensive feeding environment during the stronger selection. In addition, the number of healthy births and the rate of healthy birth of CD pigs were higher than those of AD pigs (78), which may be related to the better immunity of CD pigs. However, populations with different time dimensions (born in 2016–2017) and small sample sizes (*n* = 413–604) may affect the phenotypic comparison of the two lines. Considering the complexity of the genetic mechanisms of reproductive and immune traits, more direct reproductive and immune data are needed to validate and improve our results in the future.

### Functional-Altering Variant Likely Played an Important Role in the Breeding Process of Duroc Pigs

The Duroc pigs were mainly developed in North America but originated in Europe (17). Considering the independent domestication of Eurasian pig breeds and the loci of Eurasian wild boar populations and Chinese native pigs were all or nearly all ancestral alleles, while European domestic and commercial pigs had derived alleles, we hypothesize that the missense mutation (rs81216249) originated from European domestic pig breeds and appeared earlier than the formation of Duroc pig breeds. The DAF of Western commercial pigs was significantly higher than that of other European domestic pigs (*p* < 0.01), indicating that DAF was increased in the intensive selection processes of commercial pig breeds. This missense mutation showed very strong conservation in vertebrates and was predicted to have a deleterious effect on protein function (SIFT = 0). Previous literature demonstrated that harmful mutations are expected to be maintained at low frequency due to the efficacy of purifying selection (79). This functional-altering mutation displayed a high frequency or near fixation of derived allele in Western commercial pigs, especially Duroc pigs, revealing that this mutation may be a beneficial mutation and could improve the production traits of pigs. This speculation was further verified by the SNP–phenotypic association analysis in Sujiang pigs, a synthetic breed of Chinese and Duroc pigs, in which derived allele significantly improved the body weight and length of pigs. The derived allele also existed in other Eurasian hybrid breeds, such as Sutai and Licha pigs (49), indicating that this mutation could be used for marker-assisted hybrid breeding in Chinese native pigs. In addition, many Chinese indigenous pig breeds admixed with exotic pigs due to indiscriminate crossbreeding between Chinese and European breeds, which may lead to the loss of the original characteristics and disruption of the locally adapted gene complexes (80). This variant can also be used to detect and monitor whether Chinese local pig breeds are mixed with Western commercial pig breeds (especially Duroc pigs) and provide an effective tool for the purebred preservation of Chinese indigenous pigs. Notably, we observed only one functional-altering variant in this supergene region due to the limitation of SNP density. Considering that this missense mutation is one of the potential functional-altering mutations in this supergene and that the tested population for SNP–phenotypic association analysis was not Duroc pigs, further in-depth analyses such as resequencing, RNA-sequencing, phenotypic association studies, and functional experiments are needed to definitively determine the role of candidate genes and functional-altering mutations.

## Conclusion

In this study, we estimated the ROH patterns in two Duroc pig lines. The results showed that CD pigs had a low level of genetic diversity and a high inbreeding degree than AD pigs, which are possibly due to stronger selection. ROH hotspots revealed that a lot of shared genes putatively under selection were related to growth, sperm, and muscle development in two Duroc lines. Population-specific ROH hotspots indicated that AD may have a specific selection on female reproduction, while CD pigs may have a specific selection on immunity. Moreover, a functional-altering mutation was observed on the overlapping ROH hotspots of two Duroc populations, and the derived allele could significantly improve the body weight and length of pigs. Altogether, our results not only benefit the inbreeding management of two Duroc lines but also provide a series of promising genes that may affect economic traits in pigs.

## Data Availability Statement

The datasets presented in this study can be found in online repositories. The names of the repository and accession number can be found at: https://doi.org/10.6084/m9.figshare.8019551.v1.

## Ethics Statement

The animal study was reviewed and approved by Animal Care and Use Committee of South China Agricultural University, Guangzhou, China.

## Author Contributions

ZW and JY proposed the idea of study. XW and GL performed the analysis and wrote the manuscript. JY directed the analyses and revised the manuscript. DR, ZZ, RD, JQ, SW, YJ, JH, TG, LH, EZ, ZL, and GC collected samples and performed the experiments. ZW and JY contributed the materials. All authors contributed to the article and approved the submitted version.

## Funding

This work was supported by the Natural Science Foundation of Guangdong Province (2018B030313011), the Local Innovative and Research Teams Project of Guangdong Province (2019BT02N630), and the National High-quality Lean-type Pig Breeding United Research Program of China.

## Conflict of Interest

RD, GC, and ZW were employed by Guangdong Wens Breeding Swine Technology Co., Ltd. The remaining authors declare that the research was conducted in the absence of any commercial or financial relationships that could be construed as a potential conflict of interest.

## Publisher's Note

All claims expressed in this article are solely those of the authors and do not necessarily represent those of their affiliated organizations, or those of the publisher, the editors and the reviewers. Any product that may be evaluated in this article, or claim that may be made by its manufacturer, is not guaranteed or endorsed by the publisher.
