# Supplementary material for: Runs of Homozygosity Uncover Potential Functional-Altering Mutation Associated With Body Weight and Length in Two Duroc Pig Lines
Source: Front Vet Sci. 2022 Mar 8;9:832633. doi: 10.3389/fvets.2022.832633 (PMC8957889; doi:10.3389/fvets.2022.832633)
Supplement: Supplementary file 1 [file Data_Sheet_1.zip › Supplementary Figures.DOCX]

**Supplementary Figures**


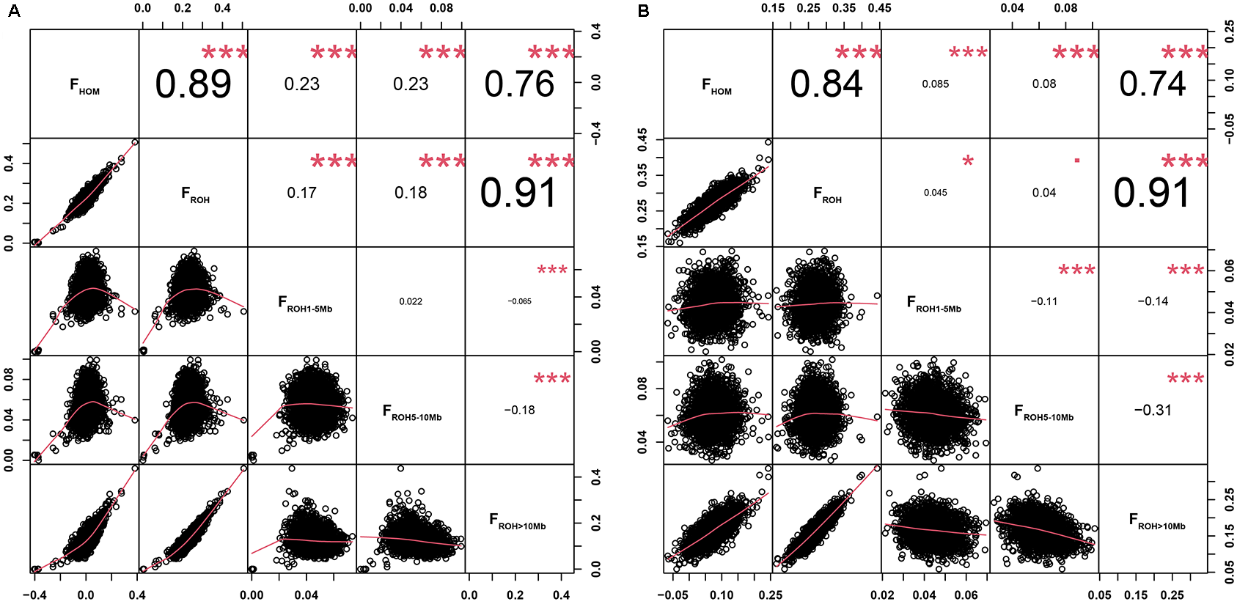


**Supplementary Figure 1**. Scatterplots (lower triangle) and correlations (upper triangle) of the genomic inbreeding coefficients between the F_HOM_ and F_ROH_ (F_ROH_, F_ROH1-5Mb_, F_ROH5-10Mb_, F_ROH >10 Mb_) in AD (A) and CD (B) pigs.


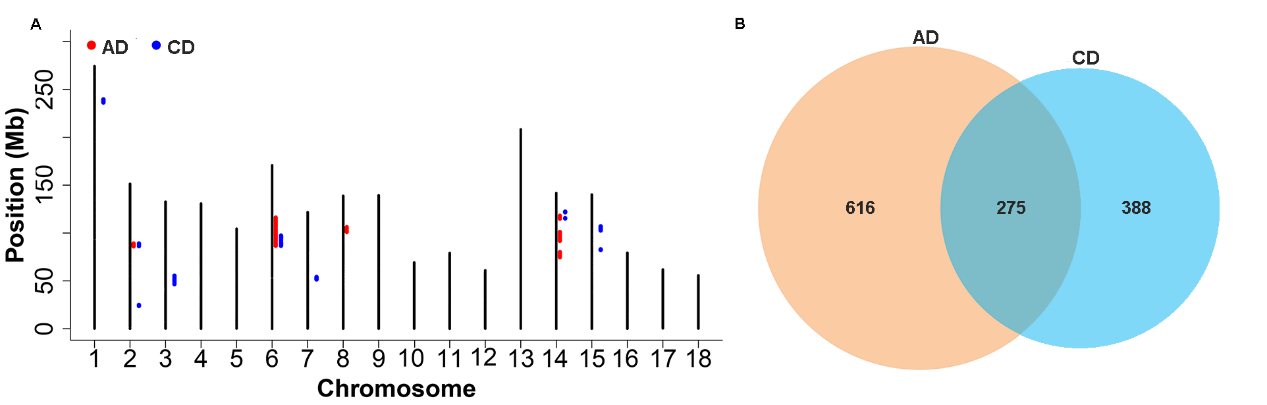


**Supplementary Figure 2.** Candidate SNPs of ROH hotspots in AD and CD pigs. (A) Genomic distribution of ROH hotspots. (B) Venn diagram of candidate SNPs in AD and CD pigs.


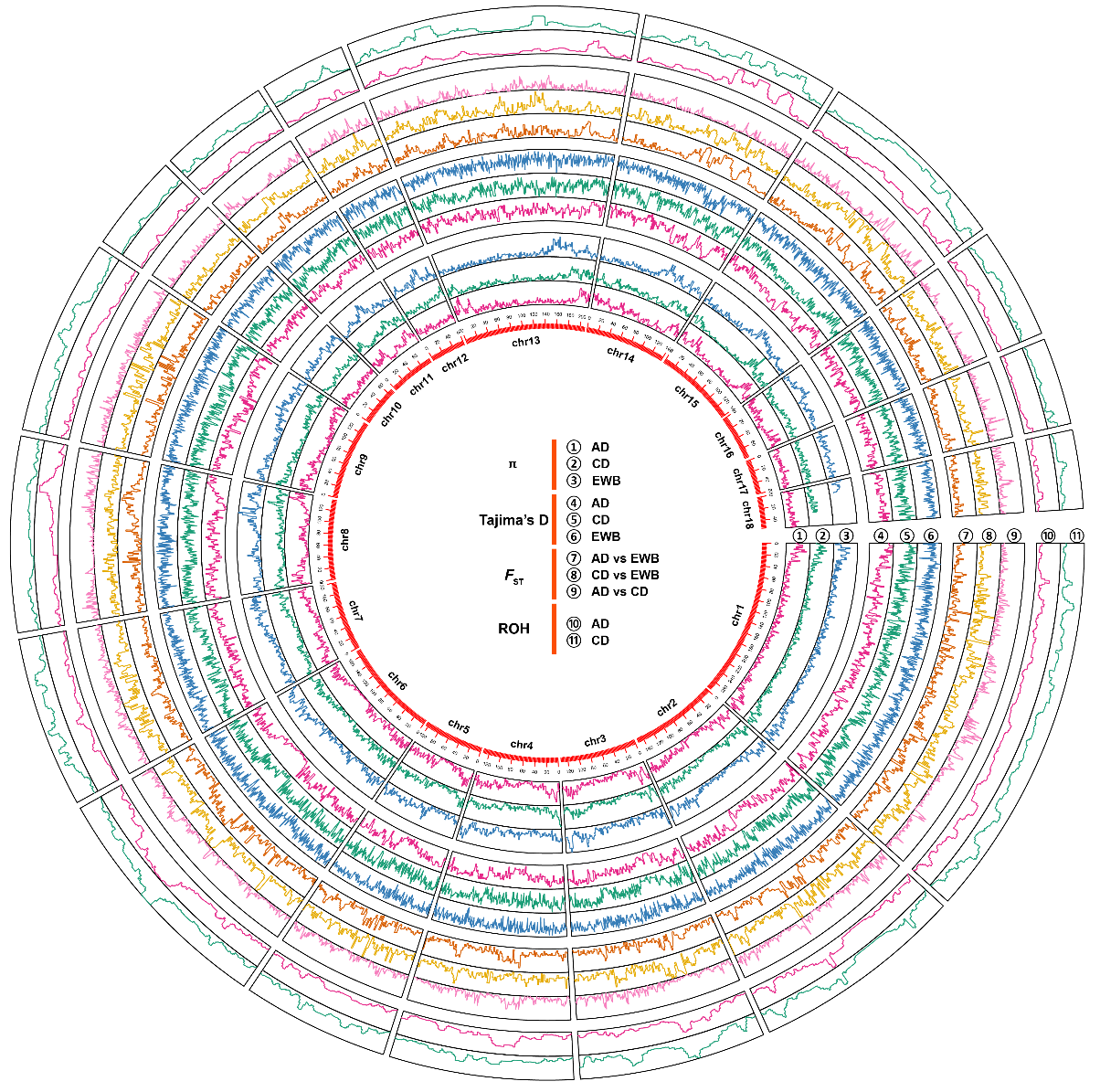


**Supplementary Figure 3.** Circos plot of ROH, π, Tajima's D and *F*_ST_ visualized by TBtools (<https://github.com/CJ-Chen/TBtools/releases>).


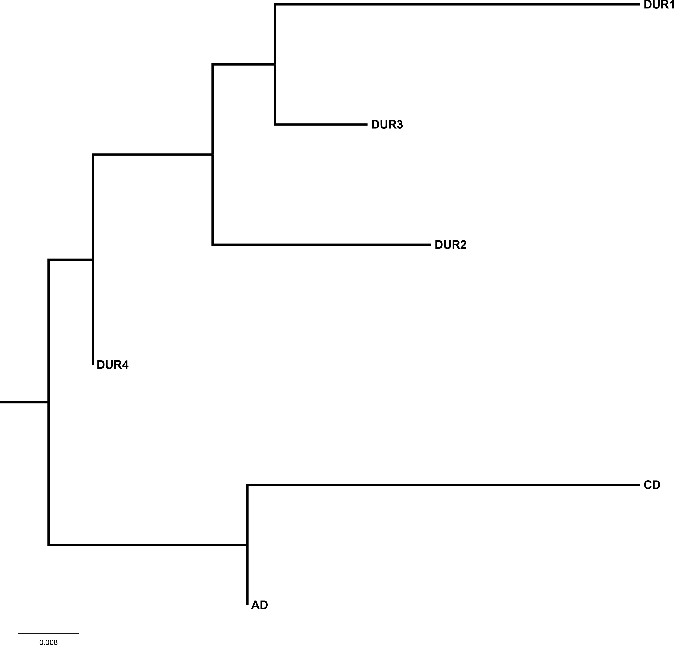


**Supplementary Figure 4.** *F*_ST_-based neighbor-joining tree of AD, CD and other 5 Duroc populations.


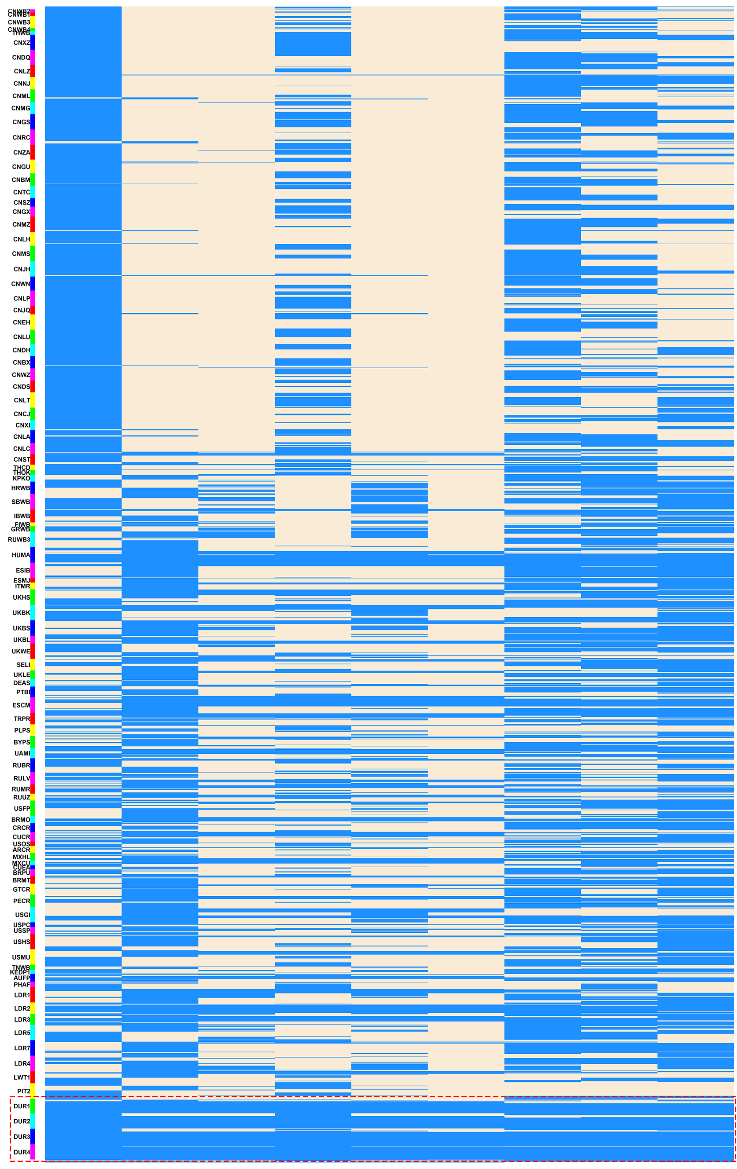


**Supplementary Figure 5.** Heat map of haplotypes within 9 SNPs regions around rs81216249.
